# Placebo for depression: we need to improve the quality of scientific information but also reject too simplistic approaches or ideological nihilism

**DOI:** 10.1186/1741-7015-12-105

**Published:** 2014-06-25

**Authors:** Andrea Cipriani, John R Geddes

**Affiliations:** 1 Department of Psychiatry, University of Oxford, Warneford Hospital, Oxford OX3 7JX, UK

**Keywords:** Antidepressants, Major depressive disorder, Meta-analysis, Placebo, Publication bias

## Abstract

The placebo response plays a major role in psychiatry, particularly in depression. A new network meta-analysis investigates whether the effects of placebo vary in studies comparing fluoxetine and venlafaxine, two widely prescribed antidepressants. Even though data from this article indicate that the effects of placebos do not differ, publication bias cannot be ruled out. The authors use their finding to criticise the paradigm of evidence-based medicine, questioning whether there is anything certain in psychiatry and, more precisely, in the field of antidepressant treatment for major depression. This study stimulates the debate about validity of scientific knowledge in medicine and highlights the importance of considering things from a different perspective. However, the authors’ view should be considered with caution. As clinicians, we make decisions every day, integrating individual clinical expertise and patients’ preferences and values with the best, up-to-date research data. The quality of scientific information must be improved, but we still think that valid conclusions to help clinical practice can be drawn from a critical and cautious use of the best available, if flawed, evidence.

Please see related articles: http://www.biomedcentral.com/1741-7015/11/230 and http://www.biomedcentral.com/1741-7015/12/106.

## Background

The placebo effect has always been an intriguing topic in medicine, probably because of both its clinical implications and its philosophical correlates for the mind-body interaction [1]. From a scientific point of view, our knowledge of the biological mechanisms for response to placebo has recently increased, thanks to more rigorous and systematic investigations [2]. Accumulating evidence suggests that the placebo effect is a genuine psychobiological phenomenon attributable to the overall therapeutic context, which includes the interaction between the patient, the clinician and the treatment environment [3].

The placebo effect has been explored across a variety of diseases and medical conditions but it is in psychiatry - and most of all in depression - that the placebo response may play a major clinical role. This has generated concerns and a long-lasting debate around the real efficacy of antidepressants, with very conflicting opinions [4,5]. In antidepressant trials with adults, the mean response rate for active treatment is 50%, while the mean placebo response is 31%, with an absolute difference of 19% [6]. This difference is even smaller if unpublished data are included in the analysis [7] or if special populations, like children and adolescents, are considered [8]. Interestingly, the placebo response has been increasing over the past 30 years and this increase does not appear to be directly explained by changes in study characteristics [6]. The real question, then, is not whether the placebo effect can influence clinical outcome but rather the extent to which it does so. A study by Naudet *et al*. published in *BMC Medicine* investigates this issue within the frame of a network meta-analysis [9].

### Study results

Accepting that randomized evidence shows both antidepressants and placebo to be effective in major depression, and that different antidepressants have different efficacy profiles, Naudet *et al*. wanted to investigate whether the effects of placebo may vary in different situations [9]. As acknowledged by the authors, this is more an epistemological question than a pragmatic clinical issue. However, Naudet and colleagues approached it scientifically and performed a review of the literature comparing the placebos of two widely prescribed antidepressants: fluoxetine and venlafaxine.

Their primary aim was to compare placebo arms in fluoxetine and venlafaxine placebo-controlled studies, so the authors looked at three different types of placebo: fluoxetine placebo (in studies where placebo was compared to fluoxetine), venlafaxine placebo (where placebo was compared to venlafaxine), and fluoxetine/venlafaxine placebo (where placebo was compared in the same trial with both venlafaxine and fluoxetine). The authors find that the two antidepressant agents are more efficacious than the placebos and the three placebos do not differ in terms of response or remission. As has been previously reported, venlafaxine is more efficacious than fluoxetine, but the funnel plots show some evidence of publication bias. Emphasising this last point - and almost neglecting the primary finding of no difference between placebos - Naudet and colleagues argue that no one can be sure placebo really equals placebo in trials of major depressive disorder. Following this theoretical reasoning, they warn clinicians to ‘step back to take a more objective view when interpreting a scientific result’, because ‘Science can never be actually sure that “sucrose = sucrose” in the treatment of major depressive disorder’. For Naudet and colleagues, these clinical implications are more important than the differences in efficacy between antidepressants (which, in our view, is much more likely and clinically very meaningful).

### Strengths and limitations of the study 

The main strength of this paper is the authors’ attempt to look at this clinical scenario in a novel way. Scientific knowledge in medicine can progress only when people try to see things from a different perspective, pursuing the truth and avoiding easy answers [10].

The primary results from this study are consistent with evidence on antidepressants in moderate to severe acute major depression: some antidepressants are more effective than placebo [11] and material differences in efficacy exist between these drugs [12]. The clinical interpretation of these findings may vary [13] and it is worth remembering that the practice of evidence-based medicine implies the integration of the best available research data with individual clinical expertise and patients’ preferences and values [14]. Evidence-based practice is not ‘cookbook’ medicine and, similarly, ranking antidepressants is not aimed at providing clinicians and patients with a list of drugs to mechanically scroll down from top to bottom. The crucial point is whether to insist on a conservative approach that considers antidepressants as a group of equivalent compounds (thus favouring the prescription of the last one put on the market) or to try to make the best use of the available evidence. We still think that valid conclusions can be drawn from a critical and cautious use of the best available, if flawed, evidence.

Currently, placebos are not an alternative for our patients when an antidepressant is indicated for moderate to severe depression. In such cases, we all prescribe an active treatment. However, when discussing the treatment plan with our patients, it would be wrong and probably clinically irresponsible to say that antidepressants are all the same and to give our patients any antidepressant, just because it is licensed and the side-effect profile is more suitable for that individual. There is evidence supporting that some drugs licensed for depression are clearly less effective than others [12] and that some do not differ from placebo [15]. Even though the decisional process varies case by case and we may end up making a completely different choice, as clinical researchers this is the best information we can give to help patients and clinicians when an antidepressant is to be prescribed for a first episode of acute major depression.

## Conclusions

### For researchers

Scientific interest is focusing on the influence of the placebo response on signal detection in clinical trials and what its physiologic mechanisms reveal about the pathophysiology of major depressive disorder [16]. High placebo response has reduced medication-placebo differences and the increasing number of failed trials has contributed to recent decisions by several pharmaceutical companies to discontinue research on medications for brain disorders [17]. The development of psychiatric medications has become progressively more time-consuming and more expensive [18]. The need for placebo superiority for regulatory approval is a matter of controversy, but it is still the rule for many regulatory agencies [19]. This is why the evaluation of future, putative antidepressant agents should require a minimisation of placebo response, the development of more efficient study designs to improve signal detection in drug development studies, and an increased antidepressant response in clinical treatment [16].

### For clinicians

In psychiatry, pharmacological treatment involves much more than simply dispensing pills, so prescribing a placebo does not mean that the patient receives no treatment at all [16]. The expectation of improvement and contact with a health-care environment with supportive and therapeutic features contribute in a probably significant proportion to the responses observed in randomised controlled trials to both medication and placebo [20]. However, these non-pharmacologic aspects of clinical management in clinical trial settings are usually not provided to the same extent in standard clinical practice. While avoiding the least effective antidepressants already available, clinicians should also combine active medication with a specific context and level of therapeutic contact, to enhance non-specific effects of treatment and gain greater treatment response.

### For study authors and journal editors

Systematic reviews and meta-analyses should be considered as scientific work, because they produce new knowledge [21] and, by definition, their findings and results are replicable. For this reason, all data should be published in the article, with figures and denominators to allow re-analysis of the data. This transparency is needed at all levels: regulatory agencies, pharmaceutical industries, scientific journals and also researchers [22]. In the review by Naudet *et al*., forest plots were originally reported without event rates, and a proper table with the characteristics of included studies and corresponding references was missing. Thanks to our commentary and the journal editor’s help, the authors have now provided the requested information and data in a comment published in *BMC Medicine*[23].

As researchers, we need to improve the quality of scientific information and keep seeking the truth; at the same time, though, as clinicians we need to reject either too simplistic approaches or ideological nihilism.

## Competing interests

The authors declare that they have no competing interests. JRG was expert witness for Dr Reddys Laboratories and is Chief Investigator on the CEQUEL trial to which GlaxoSmithKline have contributed and supplied investigational drugs.

## Authors’ information

AC is a psychiatrist who works as Senior Clinical Researcher at the Department of Psychiatry, University of Oxford. His main research focuses on the comparative efficacy of treatments, stratifying estimates for specific subgroups of patients and creating innovative methodologies for the more efficient evaluation and development of new treatments for mood disorders. In September 2013, he was appointed as Editor of the journal Evidence-Based Mental Health. JRG is Professor of Epidemiological Psychiatry and Head of the Department of Psychiatry, University of Oxford. JRG is also Director of National Institute for Health Research Oxford Clinical Research Facility and Director of the Oxford Cognitive Health and Neuroscience Clinical Trial Unit. His research focuses on the development and evaluation of treatments for people with bipolar and other mental disorders.
